# Research Progress on the Trait of Stigma Exsertion in Rice

**DOI:** 10.3390/plants13233404

**Published:** 2024-12-04

**Authors:** Hongming Guo, Yuyu Liang, Jianqun Lv, Xiangwen Su, Guangjun Ren, Fangyuan Gao

**Affiliations:** 1 Environment-Friendly Crop Germplasm Innovation and Genetic Improvement Key Laboratory of Sichuan Province, Crop Research Institute, Sichuan Academy of Agricultural Sciences (Sichuan Provincial Germplasm Resources Center), Chengdu 610066, China; hongmingguo552@163.com (H.G.); jianqunlv80@163.com (J.L.); suxiangwen_1@163.com (X.S.); 2 Key Laboratory of Tianfu Seed Industry Innovation (Co-construction by Ministry and Province), Ministry of Agriculture and Rural Affairs, Chengdu 610066, China; 3 Rice Research Institute, School of Life Science and Engineering, Southwest University of Science and Technology, Mianyang 621010, China; liangyuyu759@163.com

**Keywords:** rice, setting rate, stigma exsertion, sterility line, molecular mechanism, outcrossing habit

## Abstract

As global food demand continues to grow, enhancing rice seed-setting rate and yield has emerged as a crucial research topic. The stigma exsertion rate in rice, a pivotal determinant of the outcrossing seed-setting rate in sterility lines, is essential for facilitating the propagation and efficient seed production of hybrid rice varieties. This article reviews the research progress on stigma exertion rate in rice, systematically analyzing the latest molecular biology and genetics findings to uncover the key genes and molecular mechanisms regulating stigma exertion. Furthermore, it explores the application of molecular marker-assisted selection technology in rice breeding, aiming to optimize stigma exertion traits to enhance the stigma exertion rate and outcrossing habits of rice sterility lines. By integrating existing research outcomes, this article not only provides researchers with a theoretical foundation for a deeper understanding of the regulatory mechanisms of stigma exertion but also offers practical strategies for rice breeding practices.

## 1. Introduction

Rice is the primary food source for about half of the world’s population. However, facing the dual pressures of population growth and decreasing cultivated land, food security issues have become increasingly prominent. In recent decades, rice yields have significantly increased through the utilization of semi-dwarf alleles and the hybrid advantage [1,2]. Specifically in hybrid rice, as compared to inbred parent lines, there is a demonstrated yield advantage of 10–20%. It has been cultivated and promoted in over 40 countries worldwide, with a cumulative cultivated area exceeding 500 million hectares, resulting in a total increase in production of 6.25 million tons [3,4,5]. Despite these advancements, various challenges persist in hybrid rice production, notably its low seed production yield. To address this issue, methods such as gibberellin spraying and pollen driving have been widely used to improve seed-setting rates [6,7]. However, these measures simultaneously increase production costs. Therefore, cultivating sterile lines with high outcrossing seed-setting rates has become one of the key production methods. China and India, as the world’s largest rice producers and consumers, account for 50% of the world’s rice production and consumption. Through the research and efforts of scientists, hybrid rice has been transformed from a theoretical possibility to a reality and is now widely promoted, significantly increasing yields and making a significant contribution to global food security [1,5]. However, one of the primary obstacles to increasing seed production is the low outcrossing ability of male sterile lines. Fundamental measures to enhance seed production are comprised of improving the cultivation management of the sterile lines and modifying their outcrossing traits, such as increasing the stigma exsertion rate (SER).

In hybrid rice-breeding research, there is a significant positive correlation between the stigma exsertion rate of sterile lines and the outcrossing seed set rate. The selection of highly efficient sterile lines in breeding not only involves a comprehensive evaluation of their male sterility and combining ability but also necessitates consideration of the stigma exsertion rate. When sterile lines exhibit a higher stigma exsertion rate, the extent of stigma exsertion is increased, thereby enhancing the likelihood of contact with pollen and facilitating outcrossing. By elevating the stigma protrusion rate in male sterile lines, not only is the probability of pollen fertilization increased, but the issue of asynchrony in flowering times between parents can also be effectively addressed, thereby enhancing pollination efficiency [8]. Hence, optimizing the stigma characteristics of sterile lines during the breeding process is pivotal to enhancing the seed production yield of hybrid rice. This not only reduces the cost of seed production but also facilitates the widespread promotion of hybrid rice.

A systematic study on the variation patterns and influencing factors of stigma exsertion in rice holds profound theoretical significance and practical breeding value for elucidating the regulatory mechanisms underlying rice reproductive growth, as well as for creating hybrid rice male-sterile lines with high outcrossing ability, thereby enhancing the propagation efficiency and seed production yield of hybrid rice. This research aims to provide an overview of the latest advancements in the study of stigma exsertion in rice, and through the analysis of existing research findings, to explore its practical application value in agricultural production. Furthermore, this review intends to provide scientific theoretical underpinnings for the production practices of hybrid rice and to serve as a vital reference for further investigations in this field, thereby fostering innovation and advancement in hybrid rice technology.

## 2. Rice Stigma Exposed

**Figure 1:**
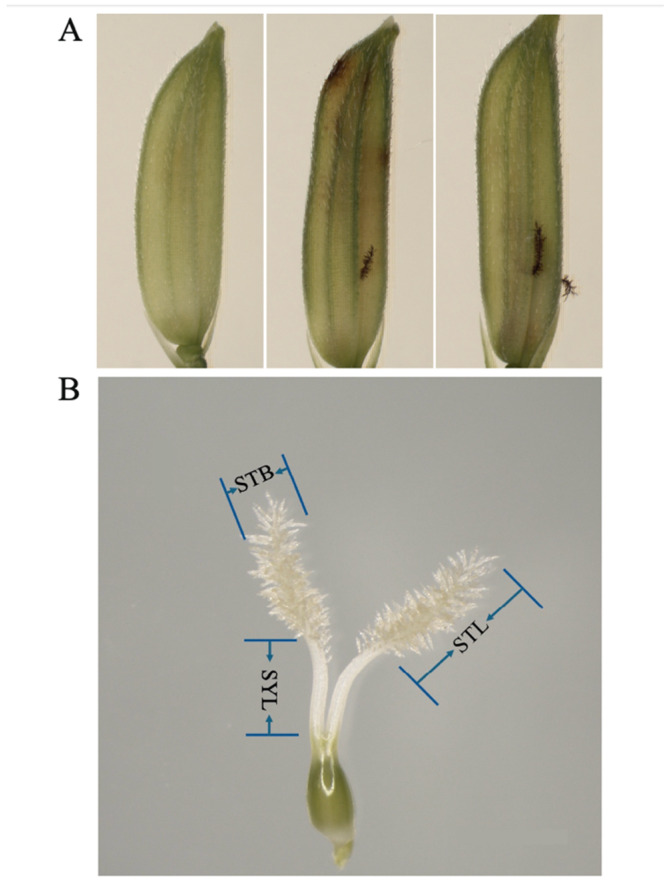
Rice stigma exsertion and related traits. (**A**). Stigma exsertion includes non-stigma exsertion, single-stigma exsertion, and dual-stigma exsertion. (**B**). Stigma-related traits. SYL—style length; STL—stigma length; STB—stigma breadth.

The phenomenon of stigma exsertion in rice refers to the stigma of the pistil remaining on the outer surface of the glumes after the glumes have finished blooming and the palea and lemma have reclosed. The stigma exsertion is further classified into single-stigma exsertion, dual-stigma exsertion, and non-stigma exsertion, based on the specific number of exposed bristles (Figure 1). Stigma exsertion is not only a crucial agronomic trait in rice, but it also serves as a significant indicator for the classification between wild and cultivated rice. In cultivated rice, the phenomenon of stigma exsertion is relatively infrequent, typically exhibiting a low rate of exsertion. This trait reveals two distinct reproductive strategies, i.e., wild rice employs outcrossing to enhance genetic diversity and adaptability to environmental variations, whereas cultivated rice maintains genetic stability and enhances yield through selfing [9]. Therefore, the changes in the physiological mechanism during the domestication process of wild rice towards cultivated rice, specifically the transition from outcrossing to selfing, possess immense research value. This shift markedly reduces the genetic segregation in the offspring of cultivated rice, profoundly impacting crop stability and yield enhancement. Concurrently, investigating the exsertion rate of stigmas holds significance for elucidating the domestication mechanisms of floral traits. In crop genetics and agricultural production practices, harnessing heterosis has been proven as one of the pivotal strategies to elevate crop yields. Nevertheless, increasing the seed production yield of hybrid rice remains a primary challenge in this field. To overcome this bottleneck, researchers are actively exploring effective methods to enhance seed production. Among these, elevating the outcrossing seed-setting rate of rice male sterile lines is considered a crucial strategy, with the exsertion rate of stigmas offering a novel research perspective. Studies indicate that a higher exsertion rate of stigmas in male sterile lines often coincides with enlarged stigma size and enhanced vigor, subsequently boosting their outcrossing ability, ultimately resulting in increased hybrid seed yields. Consequently, a highly significant positive correlation exists between the exsertion rate of stigmas and the enhancement of hybrid rice seed production yields [10,11].

## 3. Genetic Studies on Stigma Traits in Rice

The floral organ traits in rice, e.g., stigma size, anther size, and stigma exsertion rate, are quantitative traits that generally exhibit high broad-sense heritability. Studies have indicated that these traits are collectively governed by multiple QTL (quantitative trait loci), with their phenotypes displaying a normal continuous distribution, suggesting not only a rich genetic diversity but also a prominent influence by environmental factors (e.g., temperature, humidity, light intensity, and water availability). For instance, Yang et al. [12] analyzed nine floral organ traits in indica rice varieties and discovered that the heritability of stigma exsertion rate, stigma length, spikelet length, and spikelet length-to-width ratio approached or exceeded 90%, and that the heritability of anther length was also more than 80%. Furthermore, the results of studies by Li et al. [13] and Wu et al. [14] concurred with this observation, emphasizing the high heritability of most floral organ traits. Notably, in the genetic effects governing stigma exsertion rate, additive effects were the most prominent, followed by dominance and epistasis, with no evidence of cytoplasmic inheritance. Shen et al. [15] proposed that stigma exsertion rate was jointly influenced by additive, maternal, and dominant effects. Mahalingam et al. [16] conducted a study on 11 floral organ traits in five cytoplasmic male sterile lines (CMSLs) and 51 test varieties, revealing high heritability for anther length, stigma length, style width, glume opening angle, and stigma exsertion rate. Virmani and Athwal [17] analyzed the genetic effects of stigma length, determining that both dominant and additive effects were important, but the dominant effect was greater than the additive effect, and among the epistatic effects, the additive effect was the greatest. This discovery underscores the ubiquitous and relatively stable nature of high heritability in floral organ traits, which are resilient to environmental fluctuations. The research conducted by Uga et al. [18] revealed that the total length and area of rice stigmas, as well as the style, are primarily influenced by additive effects, with no maternal effects present. These research findings not only unravel the genetic potential of rice floral organ traits but also provide a robust scientific basis and abundant genetic resources for the genetic improvement of floral organ traits in hybrid breeding parents. Ultimately, they guide breeding practices and enhance breeding efficiency in rice.

## 4. Correlation of Floral Organs and Stigma in Rice

Virmani and Athwal [17] discovered a significant positive correlation between stigma exsertion rate and stigma length through detailed analysis of floral traits in 29 cultivated and wild rice species in Asia. Similarly, Uga et al. [18] utilized a recombinant inbred line population derived from the cross between the cultivated rice variety Pei-kuh and the wild rice W1944 and found that stigma exsertion was not only significantly positively correlated with stigma length, style length, glume opening angle, and length of the lemma and palea but also negatively correlated with stigma width and thickness of the lemma and palea. Li et al. [13] further validated this conclusion, noting positive correlations between stigma exsertion rate and stigma length, grain length, stigma angle, and ovary length, while observing a negative correlation with grain width. In the DH population constructed from the hybrid combination of Zhaiyeqing 8 and Jingxi 17, Yu et al. [19] found that the stigma exsertion rate was closely related to the stigma length and width and was also influenced by the length-to-width ratio of the spikelet and the length of the style. Additionally, Yan et al. [20] observed a significant positive correlation between stigma exsertion rate and both single- and double-stigma exsertion rates among 90 core germplasm resources. Moreover, Miyata et al. [21] analyzed F_2_ populations of the japonica varieties Koshihikari and IR24 and found that stigma exsertion rate was positively correlated with grain length-to-width ratio, but not significantly correlated with panicle length. These studies collectively underscore stigma exsertion as a complex phenotype influenced by multiple traits, with stigma length, spikelet length, style length, and their proportions being the primary agronomic traits contributing to an enhanced stigma exsertion rate. This finding holds significant implications for the genetic improvement of rice, facilitating the targeted selection and breeding of high-yielding rice varieties.

## 5. Interspecific Variation and Stigma Exsertion in Rice

As a naturally occurring species without artificial domestication, wild rice exhibits distinct traits during its natural selection process. It possesses large and long stigmas, as well as a high stigma exsertion rate [22]. This characteristic is particularly prominent in most wild rice germplasm, with stigma exsertion rates commonly fluctuating between 50.0% and 100.0%, significantly enhancing population reproduction and genetic diversity. In contrast, the stigma exsertion rate of African wild rice is relatively low, ranging from 3.2% to 19.7% [23]. Notably, the natural outcrossing rates of long-stamened wild rice and common wild rice approach or reach 100.0%, demonstrating their high level of outcrossing [24,25].

Compared to wild rice, cultivated rice has demonstrated diverse distribution patterns of stigma exsertion rates after prolonged periods of artificial domestication and natural evolution. Ying et al. [26] conducted analysis of 2065 rice accessions, revealing significant differences in stigma exsertion rates among various accessions. Specifically, wild rice has the highest stigma exertion rate, and African cultivated rice contains a notably greater number of accessions with high stigma exertion compared to that for Asian cultivated rice [26]. Further research has shown significant inter-subspecies differences within Asian cultivated rice, i.e., indica rice exhibits higher stigma exertion rates than does japonica rice. Among japonica rice types, tropical japonica exhibits a higher stigma exertion rate than does temperate japonica [17,27]. Additionally, Xu et al. [28] conducted a study on stigma exsertion among 435 rice varieties cultivated domestically and internationally. Their findings underscored the substantial impact of different cultivation types and geographical origins on stigma exertion rates. Notably, indica rice displayed higher stigma exertion rates than japonica rice, and terrestrial rice varieties exhibited significantly higher stigma exertion rates than aquatic rice varieties. Particularly intriguing was the observation that Yunnan Plateau japonica rice had a significantly higher stigma exertion rate than its counterparts in the Taihu Lake region [28].

In breeding practice, sterile lines and their maintainers commonly exhibit a relatively high stigma exsertion rate, a trait that varies significantly, ranging from 11.2% to 65.3% [10,29]. Notably, compared to japonica sterile lines, indica sterile lines demonstrate a more pronounced stigma exsertion rate [30,31,32]. In a specific study focusing on Thai rice germplasm, Khumto et al. [33] reported an average stigma exsertion rate of 35.6%, with extreme variations spanning from 0.0% to 75.0%, further affirming the extensive diversity of stigma exsertion traits in rice. Given that wild rice harbors superior genes for traits such as high stigma exsertion rate and large stigma, it presents significant potential for genetic improvement of stigma exsertion traits and germplasm resource innovation [34]. Therefore, future breeding research may consider integrating molecular-marker-assisted technology with traditional breeding methods, aiming to introduce elite genes from wild rice into sterile line hybrid seed production, thereby enhancing hybrid seed yield.

## 6. Identified QTL for Stigma Exsertion in Rice

The mapping populations employed for the localization of QTLs related to stigma exsertion exhibit considerable diversity and complexity, encompassing a broad spectrum of genetic resources such as the recombinant inbred line (RIL), near isogenic line (NIL), doubled haploid (DH) populations, F2 segregating populations, backcross populations (BC), chromosome segment substitution line (CSSL), cytoplasmic male sterile line (CMSL), association mapping population (AMP), single-segment substitution line (SSSL), and secondary SSSL (s-SSSL). The genetic architecture of these populations significantly impacts the precision of quantitative trait localization. To date, researchers have identified over 100 QTLs associated with unilateral, bilateral, and total stigma exsertion in rice that are extensively distributed across 12 chromosomes, with variations observed in their localization outcomes. The majority of these QTLs originate from indica maintainers or sterile lines, wild rice, and indica subspecies among conventionally cultivated rice (Table 1). Given that stigma exsertion is a trait governed by multiple minor-effect genes and is highly susceptible to environmental fluctuations, most of QTLs contribute little to the phenotype. Nevertheless, it is noteworthy that a few QTLs demonstrate notable contributions. For instance, Miyata et al. [21] identified *qES3* on chromosome 3 within the D83726-T86 marker interval, with a remarkable contribution rate of 32%. Furthermore, Li et al. [35] localized *qPES-9* between the RM105 and RM566 markers on chromosome 9, which exhibited an exceptionally high contribution rate of 76.6%. In contrast, the rest of the identified QTLs had a contribution rate of around 10%.

The positioning populations, marker densities, and analytical methods employed by different scholars vary significantly, leading to the identification of numerous and complex QTLs that are difficult to directly summarize into general patterns. However, overlaps between some QTLs have been observed. For instance, the *qPES-1* identified by Li et al. [44] overlaps with the *qTSE-1* identified by Li et al. [50] within the RM1247-RM7383 interval. Similarly, the *qPES-2* identified by Li et al. [37] and Deng et al. [42] on chromosome 2 also exhibits an overlap within the RM1285-G1327 interval. Furthermore, the *qPES-3* reported by Qiao et al. [40] overlaps with the *qTSE-3a* identified by Li et al. [50]. Zou et al. [66] utilized single-segment substitution lines (SSSLs) derived from three AA-genome wild rice species (*O. barthii*, *O. meridionalis*, and *O. rufipogon*) to identify a total of 36 QTLs related to stigma exsertion on 11 chromosomes. By comparing the chromosomal intervals of these QTLs with previously reported examples, they found that 12 were novel, while 24 were either identical or overlapped with previously reported QTLs [66]. Notably, the chromosomal segment covered by *qSERb3-1* coincides with the *qES3* region identified by Miyata et al. [21] using a segregating population derived from Koshihikari and IR24 [66].

Further analysis of QTLs jointly identified by multiple researchers, focusing on their overlapping or covered regions, and the subsequent implementation of fine mapping hold significant academic value. These QTLs exhibit notable differences in their contribution rates to stigma exsertion, which could stem from various factors such as differences in mapping populations, the choice and density of marker types, as well as factors intimately tied to the researchers’ threshold settings and the genetic backgrounds of the parental lines used in QTL identification. Given the intricate interplay between stigma exsertion and numerous floral traits, along with environmental factors, the fine mapping of this trait and its subsequent applications face numerous challenges. Consequently, breeding research urgently necessitates broadening the scope of exploration and actively tapping into rice germplasm resources with high stigma exsertion characteristics.

## 7. Localization of QTL for Other Traits in Rice Stigma

Numerous studies have revealed a significant positive correlation between the stigma exsertion rate of rice and its stigma length (STL) and stigma width (STB), as well as its style length (SYL) [17,76]. The stigma length, stigma width, and style length of rice are relatively less influenced by environmental factors and are primarily quantitative traits controlled by multiple genes. Increasing the length and width of stigmas, as well as the length of styles, can enhance the outcrossing seed-setting rate of male sterile lines, thereby improving the yield of hybrid seed production. The research on the stigma length, stigma width, and style length traits among rice stigma characteristics has been relatively late compared to that regarding the stigma exsertion rate, and thus, there are fewer QTLs identified for these traits. The reported QTLs are distributed across 12 chromosomes (Table 2). Uga et al. [77] performed QTL analysis for stigma-related traits using five mapping populations (including Milyang23/Akihikar RILs, Asominori/IR24 RILs, Nipponbare/Kasalath BC populations, IR64/Azucena DH populations, and IR64/Kinandang Patong F2 populations), identifying 18 QTLs for stigma length, 15 for stigma width, and 10 for style length. Beyond traditional mapping methods, researchers have also employed genome-wide association studies (GWASs) to locate QTLs for rice stigma traits. For instance, Yan et al. [20] identified two QTLs associated with stigma length through GWAS using 90 mini-core collections from the United States Department of Agriculture. Dang et al. [78] conducted a GWAS on 227 rice varieties, identifying six QTLs related to stigma length. Additionally, Marathi et al. [22] performed SNP genotyping on 48 cultivated and wild rice varieties, successfully identifying six QTLs related to stigma length on chromosomes 3, 4, 7, and 10. Interestingly, some studies have found that the intervals of QTLs for certain traits in rice, such as stigma length and style length, overlap with the intervals of QTLs for stigma exsertion rate. For example, *qPES-10*, *qSTL-10*, and *qTSSL-10* identified on chromosome 10 overlapped in the interval RM171-RM1108, as determined by Jiang et al. [67]. Therefore, studying the QTLs that control these traits is of great significance. By localizing the genes that control various stigma traits, we can significantly enhance the improvement of the outcrossing characteristics of male sterile lines.

## 8. Gene Cloning for Stigma Exsertion in Rice

The study of the stigma exsertion trait in rice, as a complex genetic characteristic co-regulated by multiple genes, has produced significant scientific progress in regards to gene cloning, functional verification, and its genetic regulatory mechanisms in recent years. Extensive research has revealed a shared genetic regulatory network between stigma exsertion and grain shape traits in rice. These genes can directly or indirectly affect grain shape or stigma size by delicately regulating cellular development processes, ultimately influencing the extent of stigma exsertion.

Zhou et al. [57] conducted a genome-wide association analysis utilizing approximately 6.5 million single nucleotide polymorphisms (SNPs) across 533 distinct rice cultivars aimed at investigating the characteristics of stigma exsertion and related floral organs. The analysis successfully identified 23 genomic regions significantly associated with stigma exsertion and its correlated traits. Notably, three loci were found to be tightly co-localized with the major grain shape genes *GS3*, *GW5*, and *GW2*. Furthermore, research by Zhu et al. [83] demonstrated that simultaneous knockout of three grain shape genes, *GS3*, *GW8*, and *GS9*, led to a remarkable enhancement in the length-to-width ratio of spikelets, as well as increased stigma and style lengths, thereby increasing the rate of stigma exsertion without adversely affecting other agronomic traits. Specifically, in the japonica rice variety Hua11 and the indica male-sterile line Zhu6S, precise manipulation of these grain morphology genes significantly improved both stigma exsertion and outcrossing rates [83].

Miyata et al. [21] named a main effector QTL detected on chromosome 3 as qES3, which was later verified to be the cloned gene *GS3*. [47]. A nonsense mutation in the second exon of *GS3* results in an increase in the number of cells in the non-bristled region of the stigma, thereby enhancing stigma length and facilitating stigma exsertion [47]. Through GWAS of 533 diverse rice varieties, Zhou et al. [57] revealed that the influence of the *GS3* on stigma length is significantly more pronounced in indica rice compared to japonica rice. In the indica cultivar Minghui 63, the overexpression of *GS3* in negative transgenic plants exhibited a stigma exsertion rate that was 66% to 76% higher than that in positive plants. Furthermore, *GS3* overexpression led to a reduction in style length and an increase in stigma width, imparting a shorter and wider characteristic to the stigma morphology [57].

The *GW5* gene is a major QTL controlling grain width and grain weight in rice, while also exerting a notable influence on stigma exsertion [84]. In the japonica rice variety Kongyu 131, the overexpression of *GW5* markedly enhanced the length and width of the style and stigma, resulting in a 17–24% increase in the stigma exsertion rate compared to that of the control plants. This process primarily occurs through *GW5* altering the number of glume cells vertical to the style, rather than directly acting on the length of the style or stigma itself [57]. *GW2* is another key QTL that affects both grain width and 1000 grain weight in rice [85]. In the japonica rice variety Zhonghua 11, when *GW2* expression is suppressed, the stigma length, width, and style length of the plants all significantly increase, ultimately elevating the stigma exsertion rate by 5–10% [57].

*qSTL3* is another well-known stigma exsertion gene. Liu et al. [78] constructed a CSSL using Kasalath as the donor and Nipponbare as the recipient. They discovered that there were significant differences in stigma length and exsertion rate between SSSL14 and Nipponbare. The QTL that controls stigma length was designated as qSTL3. Subsequently, an F_2_ population was generated through the crossing of SSSL14 with Nipponbare, and *qSTL3* was fine-mapped to a 19.8 kb region containing three genes. Further analysis revealed a polymorphism in the gene *LOC_Os03g14850* between Kasalath and Nipponbare. Experimental validation using a T-DNA insertion mutant showed that the mutant exhibited an 8.62% increase in stigma length compared to that of the wild type, indicating that this gene is a negative regulator of stigma length. Additionally, this gene also significantly influenced grain length in rice, revealing its potential pleiotropic effects [78].

In addition to genes associated with grain shape, there are many genes that are closely related to rice stigma traits and their development. For example, Dang et al. [86] found that the *qSYL3-k* gene, which belongs to the MADS-box family of transcription factors, had a significant effect on style length by map cloning. The *qSYL3-k* gene extends the overall length of the style by increasing the length of the cells within the style, a process closely associated with elevated levels of GA4 in the pistil. Analysis of the *qSYL3* locus across 136 varieties revealed that the *qSYL3^AA^*, *qSYL3^AG^*, and *qSYL3^GA^* haplotypes of the alleles contribute to an increase in style length, whereas the *qSYL3^GG^* haplotype leads to a reduction [86]. Furthermore, Guo et al. [70] successfully constructed NIL using the japonica rice male sterile line DaS, with high stigma exsertion, and D50, with low stigma exsertion. They localized *qSE4* to a specific region on chromosome 4. Their investigation revealed a nucleotide substitution in the promoter region of the *ARF10* gene between DaS and D50. Further experimentation demonstrated that knockout of the *ARF10* in the DaS background resulted in *arf10* mutants with significantly lower stigma exsertion rates than those of the wild type, accompanied by significant changes in auxin content and the expression of auxin signaling-related genes [70]. Moreover, the overexpression of miR167d resulted in obstructed elongation of the filaments, enlarged stigmas, and altered lemma morphology. Its target genes, *ARF6*, *ARF12*, *ARF17*, and *ARF25*, exhibited overlapping functions in flower opening and stigma size regulation. Specifically, any single mutation in *ARF12* combined with mutations in *ARF6*, *ARF17*, or *ARF25* presented defective phenotypes reminiscent of miR167d overexpression [87]. Although some QTLs or genes associated with stigma traits have been finely mapped or cloned, the underlying genetic mechanisms remain incompletely understood. Current research on rice stigma exsertion primarily remains at the preliminary mapping stage, necessitating further precise mapping of overlapping or co-localized QTLs and early identification of target genes through genetic experiments to advance functional analyses.

## 9. Breeding Utilization of QTLs for Rice Stigma Exsertion

In the field of crop genetic improvement, traditional breeding methods mainly rely on techniques such as hybridization, backcrossing, and recrossing, combined with external phenotypic selection aimed at improving the genetic characteristics of plants. For the use of major QTL for controlling stigma exsertion, hybridization and multi-generation backcrossing techniques, combined with phenotypic selection, can be used to introduce the primary QTL into materials with low stigma exsertion, thereby improving their stigma exsertion. For example, Li et al. [49] successfully introduced the *qSe1* gene, which regulates stigma exsertion rate in Chinese aromatic rice, into the parent of Chuanxiang 29B, which significantly improved the stigma exsertion rate to 16.4%. On the other hand, Li et al. [88] significantly improved the traits of the low-stigma-exsertion-rate maintenance line Hukuang 1B through hybridization and multi-generation backcrossing using the high-stigma-excursion-rate maintenance line K17B as a donor and further transferred the line through backcrossing to produce an improved sterile line with a stigma exsertion rate of greater than 60.0%.

In addition, important progress has been made in the research of Wu et al. [89], who successfully introduced QTLs for high stigma exsertion rate in Yue Tai and II-32B into maintenance lines with low stigma exsertion rate, which not only improved the stigma exsertion rate of the maintenance and sterile lines, but also enhanced the seed production yield of hybrid rice through a series of improvement measures. However, it is important to note that unfavorable agronomic traits in many germplasm resources tend to be closely interlinked with high-stigma-exsertion-rate traits. Long-term practice has shown that molecular marker-assisted selection (MAS) is an effective means to improve breeding efficiency. The fundamental principle of MAS is to use molecular markers that are closely linked or cosegregated with the target gene, enabling the early-stage screening of target traits controlled by these genes during hybridization. This approach minimizes linkage drag, expedites the acquisition of desired individuals, and consequently, significantly elevates breeding efficiency [90]. For instance, Miyata et al. [21] successfully introgressed the allele *qES3*, which notably enhances stigma exsertion rate in IR24, into Koshihikari through MAS, resulting in a 36% increase in stigma exsertion rate. Similarly, Wang et al. [8] also employed MAS to integrate multiple QTLs for high stigma exsertion rate from indica rice into japonica rice maintainer lines, significantly elevating the stigma exsertion rate of japonica sterile lines from an initial level below 30.0% to over 80.0%. Moreover, Cai et al. [91] utilized QTLs (*qPES3*, *qPES9*, and *qPES12*) associated with high stigma exsertion rate from the indica sterile line 50S. Through a combined approach of hybridization, backcrossing, and marker-assisted selection, these QTLs were introgressed into japonica rice maintainer lines, leading to a 30–60% increase in stigma exsertion rate in the introgressed lines compared to that in the control group [91].

## 10. Future Perspectives

With the in-depth exploration of the mechanisms underlying the differences in stigma exsertion between the indica and japonica subspecies of rice, particularly the elucidation of the mechanisms by which grain shape and stigma shape influence stigma exsertion, breeding scientists will be able to more precisely manipulate the relevant genes to develop hybrid rice varieties that exhibit both high yield and high quality. This involves integrating QTLs related to stigma exsertion from diverse genetic backgrounds, such as *GW2*, *GS3*, *GW5*, *GW8*, and *GS9*. Studies have shown that the pyramided genotype PL-*gs3*/*GW7*/*gw8* exhibits the highest ratio of grain length to width (RLW) and optimal stigma exsertion, highlighting its tremendous potential as an excellent breeding material [92]. Therefore, by pyramiding QTLs that regulate stigma exsertion with key genes controlling elongated grain shape, it is promising to create high-quality male-sterile lines with significantly improved stigma exsertion, pointing to a new direction in rice breeding.

Furthermore, the continuous advancement of gene editing technologies, especially the widespread use of tools like CRISPR/Cas9, has made the precise regulation of specific genes possible. This not only enables the validation of current theoretical hypotheses regarding the impact of grain shape and stigma shape on stigma exsertion but also opens up a new path for developing high-SER rice varieties through molecular design breeding approaches.

In summary, future research on rice genetic breeding aimed at enhancing stigma exsertion will increasingly the focus on the integration and innovative utilization of gene resources, as well as the precise application of gene editing technologies. Through interdisciplinary collaboration and technological innovation, it is possible to achieve breakthrough improvements in rice stigma exsertion, thereby making significant contributions to global food security and sustainable agricultural development.

## Figures and Tables

**Table 1:** Previously identified QTL for stigma exsertion in rice.

Source of Mapping Population	Population	Chromosomes	QTLs	References
T821B/G46B	F2	Chr. 1, 2, 3, 7, 9	*qPES-1*, *3*, *7*, *9*, *qPDES-2*, *9*, *qPSES-1-1*, *1-2*, *9*	[35]
Aijiao Nante/P16	F2	Chr. 6	*es-1*	[36]
Dongxiang/Guichao2	BC1	Chr. 5, 8	*qPEST-5*, *8*	[37]
Pei-kuh/W1944	RIL	Chr. 5, 10	*qRES-5*, *10*	[18]
Zaiyeqing8/Jingxi17	DH	Chr. 2, 3	*qPES-2*, *3*	[38]
Asominori/IR24	RIL	Chr. 3, 4, 6, 8	*R1002*, *C1468*, *XNpb238*, *R1468B*, *XNpb331*, *C1003B*, *C227*	[39]
Zhenshan97B/IRAT109	RIL	Chr. 1, 2, 4, 5, 8, 9, 12	*qPSES-1*, *2*, *5*, *10*, *12*, *qPDES-1a*, *1b*, *2*, *4*, *5*, *8*, *9*, *qPES-1a*, *1b*, *2*, *5*, *9*, *12*	[40]
Nipponbare/Kasalath	BIL	Chr. 3, 4, 5	*qPES-3*, *4*, *5*	[41]
Hoshinohikari/IR24	F2	Chr. 3	*qES3*	[21]
90 accessions	AMP	Chr. 1, 5, 6, 7, 8, 9, 10, 11	*qSSE-1*, *6*, *9*, *10*, *qDSE-1*, *5*, *7*, *8*, *11*, *qPES-5*, *7*, *8, 9*, *10*	[20]
Huhan1B/Ⅱ-32B	F2	Chr. 3, 4, 7, 9	*qPSES-3*, *7*, *9*, *qPDES-3*, *9*, *qPES-3*, *4*, *7*, *9*	[42]
Nuo5/YouⅠB	F2	Chr. 2, 5, 8	*qPES-2*, *5*, *8*, *qPES-2*, *5*, *8*	[43]
50S/LianB	F2	Chr. 3, 9, 12	*qSPES3*, *qPES-3*, *9*, *12*	[44]
60B/Liaojing9	F2	Chr. 1, 2, 5, 8, 9	*qPGCS-1*, *8*, *qPGCD-8*, *9*, *qPGCES-2*, *8*	[45]
II-32B/G46B	F2	Chr. 1, 2, 5, 8	*qPES1*, *2*, *5*, *8*	[46]
IR24/Asominori		Chr. 3	*GS3*	[47]
Yuezaoxian6/II-32B	RIL	Chr. 1, 3, 5, 6, 7, 9	*qPDES-1*, *3*, *6*, *7-1*, *7-2*, *9-1*, *9-2*, *9-3*, *qPSES-1*, *5*, *6*, *7*, *9-1*, *9-2*, *9-3*	[48]
ZX/CX29B	RIL	Chr. 1, 3, 6, 7, 9, 10, 12	*qDSE-1*, *6a*, *6b*, *10*, *qSSE-1*, *3*, *6a*, *6b*, *7*, *9*, *12*	[49]
Zhenshan97B/9311	BIL	Chr. 1, 2, 3, 5, 7, 8, 9	*qSSE-1*, *5*, *9*, *qDSE-1*, *3*, *5*, *7*, *8*, *qTSE-1*, *2*, *3a*, *3b*, *5a*, *5b*, *7*, *8*, *9*	[50]
HuhanlB/K17B	F2	Chr. 5, 6, 7	*qPSES-5*, *qPDES-5*, *6*, *7*, *qPES-6*	[51]
XieqingzaoB/Zhonghui9308	RIL	Chr. 1, 6, 10, 11	*qSSE-6*, *11*, *qDSE-1a*, *1b*, *10*, *11*, *qTSE-1*, *11*	[52]
217 CMS line	AMP	Chr. 3, 5, 6, 8, 10, 11, 12	*qDSE-5*, *8*, *10*, *11*, *qTSE-3.1*, *3.2*, *6.1*, *6.2*, *8.1*, *8.2*, *8.3*, *11*, *12*	[53]
XieqingzaoB/Zhonghui9309	NIL	Chr. 11	*qSSE11*, *qDSE11*, *qTSE11*	[54]
XieqingzaoB/Zhonghui9308	CSSL	Chr. 5, 6, 10, 11	*qSSE-5*, *10*, *11*, *qDSE-10*, *11*, *qTSE-5*, *6*, *10*, *11*	[55]
DaS/D50	F2	Chr. 1, 2, 3, 4, 6, 7, 12	*qPSES-1*, *2*, *3.1*, *3.2*, *4*, *12*, *qPDES-1*, *2.1*, *2.2*, *4*, *6.1*, *6.2*, *12*, *qPES-2.1*, *2.2*, *3*, *4*, *6.1*, *6.2*, *7*, *12*	[56]
533 accessions	AMP	Chr. 2, 3, 5, 8, 9	*qSSE-3*, *5*, *9*, *qDSE-5*, *8*, *qTSE-2*, *3*, *5*	[57]
115S/93S	F2	Chr. 10	*qLESR10*	[58]
Gui 2136S/Nipponbare	F2	Chr. 3	*qPES-3*, *qPDES-3*, *qPES-3*	[59]
XieqingzaoB/Zhonghui9308	CSSL	Chr. 7	*qSSE7*, *qDSE7*, *qTSE7*	[60]
Akidawara/W0120	F2	Chr. 3, 8	*qSER-3*, *8*	[61]
Huhan1B/II-32B	NIL	Chr. 7	*qSER-7*	[62]
ZS616/DS552	F3	Chr. 3, 4, 5, 6, 8, 11	*qSER-3.1*, *3.2*, *4.1*, *5.1*, *6.1*, *8.1*, *11.1*	[63]
58B/Nipponbare	F2	Chr. 1, 2, 3, 4, 5, 7, 8, 10, 12	*qSPES-1*, *2*, *3*, *4*, *10*, *qDPES-1*, *2*, *5*, *8*, *qPES-2*, *7*, *8*, *12*	[64]
IRGC104387	SSSL	Chr. 1, 3, 5, 9, 10	*qSER-1a*, *1b*, *3a*, *3b*, *5*, *9*, *10*	[65]
HJX74/O. rufipogon	SSSL	Chr. 3, 5, 6, 8, 10, 12	*qSERb3-1*, *5-1*, *6-1*, *8-1*, *12-1*, *qSERm5-1*, *6-1*, *8-1*, *10-1*	[66]
Xiushui79/C Bao	RIL	Chr. 1, 6, 8, 9, 10, 12	*qPES-1*, *6.1*, *6.2*, *8*, *9*, *10*, *12.1*, *12.2*	[67]
IR66897B	SSSL	Chr. 2, 3	*qSER-2a*, *2b*, *3a*, *3b*	[68]
1892S/Yangdao6-xuan	RIL	Chr. 1, 2, 3, 4, 5, 7, 8	*qSSE-1*, *2-1*, *2-2*, *4*, *5*, *7*, *8*, *qDSE-1*, *3*, *4*, *7*, *qTSE-1*, *2*, *3*, *4*, *7*	[69]
DaS/D50	NIL	Chr. 4	*qSE4*	[70]
Zhenshan 97B/IRAT109	RIL	Chr. 1, 2, 8	*qSSE-1*, *2*, *8*, *qDSE-1*, *8*, *qTSE-1*, *2*, *8*	[71]
O. glaberrima	SSSL	Chr. 1, 3, 5, 8, 12	*qSER-1a*, *1b*, *3*, *5*, *8a*, *8b*, *12*	[72]
HJX74	SSSL	Chr. 3	*qSER3a-sat*	[73]
02428/ZH464	F2	Chr. 2, 4	*qTSE-2*, *4*	[74]
SG22/HJX74	s-SSSL	Chr. 1	*qSERg-1b*	[75]

Note: ES, PEST, RES, PES, PGCES, and TSE represent exserted stigma. PSES, SSE, SPES, and PGCS represent percent of single-stigma exsertion. PDES, PGCD, DSE, and DPES represent percent of double-stigma exsertion. LESR represents low-exposed stigma.

**Table 2:** Previously identified QTL for stigma length, width, and style length in rice.

Source of Mapping Population	Population	Chromosomes	QTLs	References
Pei-kuh/W1944	RIL	Chr. 4, 6, 12	*qSTL-4*, *6*, *qSTB-4*, *12*, *qSYL-6*	[18]
90 accessions	AMP	Chr. 3, 10	*qSTL-3*, *10*	[20]
T821B/G46B	F2	Chr. 1, 2, 3, 7, 9	*qSTB-6*, *12*, *qSTL-3*, *6*, *7*, *9-1*, *9-2*, *qSYL-3*, *6*	[44]
Milyang23/Akihikari	RIL	Chr. 1, 2, 3, 4, 6, 7, 10, 12	*qSTL-1*, *3*, *10*, *12*, *qSTB-1*, *2*, *3*, *4*, *6*, *12*, *qSYL-1*, *4*, *7*	[77]
Asominori/IR24	RIL	Chr. 3, 4, 5, 7, 12	*qSTL-3-1*, *3-2*, *7*, *12*, *qSTB-3*, *5*, *7*, *qSYL-3*, *4*	[77]
Nipponbare/Kasalath	BIL	Chr. 2, 3, 7	*qSTL-2*, *3*, *qSTB7*,	[77]
IR64/Azucena	DHL	Chr. 1, 2, 3, 5, 9	*qSTL-3*, *5*, *9*, *qSTB-3*, *5*, *9*, *qSYL-1*, *2*, *3*	[77]
IR64 · Kinandang Patong	F2	Chr. 1, 2, 3, 5	*qSTL-3-1*, *3-2*, *3-3*, *5-1*, *5-2*, *qSTB-2*, *3*, *qSYL-1*, *3*	[77]
227 accessions	AMP	Chr. 1, 2, 4, 6	*qSTL-1*, *2-1*, *2-2*, *4*, *6-1*, *6-2*	[78]
Nipponbare/Kasalath	CSSL	Chr. 3	*qSTL3*	[79]
48 accessions	AMP	Chr. 3, 4, 7, 10	*qSTL-3-1*, *3-2*, *qSYL10*, *qSSL-3*, *7*	[22]
Nipponbare/W630	BRIL	Chr. 7, 8	*qSGL-7*, *8*, *qSYL8*	[80]
533 accessions	AMP	Chr. 3, 4, 8	*qSTL8*, *qSYL-3*, *4*	[57]
SPR1/O. rufipogon Griff	BIL	Chr. 3, 8, 10	*qSTL-3A*, *3S*, *8A*, *8S*, *10S*, *qSTW-3S*, *8S*, *10S*, *qSTYL-8A*, *10S*	[81]
Xiushui79/C Bao	RIL	Chr. 1, 2, 3, 6, 7, 9, 10, 11, 12	*qSTL-2*, *3*, *6*, *7*, *9*, *10*, *11*, *12*, *qTSSL-1*, *2.1*, *2.2*, *3*, *7*, *9*, *10*, *11*, *12*	[67]
IR64/OL	BIL	Chr. 1, 2, 3, 5, 8, 11	*qSTGL-2-1*, *5-1*, *8*, *11-1*, *11-2*, *qSTYL-1-1*, *5-2*, *8-1*, *qSTGB-1-1*, *3-1*	[82]

Note: STL, SGL, and STGL represent stigma length. STB, STW, and STGB represent stigma width. SYL and STYL represent style length. SSL and TSSL represent the sum of stigma and style length.
